# Pan-Genome of Novel *Pantoea stewartii* subsp. *indologenes* Reveals Genes Involved in Onion Pathogenicity and Evidence of Lateral Gene Transfer

**DOI:** 10.3390/microorganisms9081761

**Published:** 2021-08-18

**Authors:** Gaurav Agarwal, Ronald D. Gitaitis, Bhabesh Dutta

**Affiliations:** Department of Plant Pathology, Coastal Plain Experiment Station, University of Georgia, Tifton, GA 31793, USA; dronion@uga.edu

**Keywords:** pangenome, horizontal gene transfer (HGT), core genome, accessory genome

## Abstract

*Pantoea stewartii* subsp. *indologenes* (*Psi*) is a causative agent of leafspot on foxtail millet and pearl millet; however, novel strains were recently identified that are pathogenic on onions. Our recent host range evaluation study identified two pathovars; *P. stewartii* subsp. *indologenes* pv. *cepacicola* pv. nov. and *P. stewartii* subsp. *indologenes* pv. *setariae* pv. nov. that are pathogenic on onions and millets or on millets only, respectively. In the current study, we developed a pan-genome using the whole genome sequencing of newly identified/classified *Psi* strains from both pathovars [pv. *cepacicola* (*n* = 4) and pv. *setariae* (*n* = 13)]. The full spectrum of the pan-genome contained 7030 genes. Among these, 3546 (present in genomes of all 17 strains) were the core genes that were a subset of 3682 soft-core genes (present in ≥16 strains). The accessory genome included 1308 shell genes and 2040 cloud genes (present in ≤2 strains). The pan-genome showed a clear linear progression with >6000 genes, suggesting that the pan-genome of *Psi* is open. Comparative phylogenetic analysis showed differences in phylogenetic clustering of *Pantoea* spp. using PAVs/wgMLST approach in comparison with core genome SNPs-based phylogeny. Further, we conducted a horizontal gene transfer (HGT) study using *Psi* strains from both pathovars along with strains from other *Pantoea* species, namely, *P. stewartii* subsp. *stewartii* LMG 2715^T^, *P. ananatis* LMG 2665^T^*, P. agglomerans* LMG L15, and *P. allii* LMG 24248^T^. A total of 317 HGT events among four *Pantoea* species were identified with most gene transfer events occurring between *Psi* pv. *cepacicola* and *Psi* pv. *setariae*. Pan-GWAS analysis predicted a total of 154 genes, including seven gene-clusters, which were associated with the pathogenicity phenotype (necrosis on seedling) on onions. One of the gene-clusters contained 11 genes with known functions and was found to be chromosomally located.

## 1. Introduction

The *Pantoea* complex is constituted by four species, namely, *P. ananatis*, *P. stewartii*, *P. allii* and *P. agglomerans* that cause center rot of onions [1,2,3,4]. Three out of the four species in the *Pantoea* species complex (*P. ananatis, P. agglomerans* and *P. stewartii* subsp. *indologenes*) are responsible for more than 80% of the reported cases of disease in onions [5]. Earlier, Mergaert et al. [6] reclassified *Eriwinia stewartii* as *P. stewartii* and proposed two subspecies namely, *P. stewartii* subsp. *stewartii* (*Pss*) and *P. stewartii* subsp. *indologenes* (*Psi*). Recently, we phenotypically and genotypically characterized seventeen *Psi* strains that are either pathogenic on both onions and millets or on millets only [7]. Based on a host-range evaluation, we proposed two new pathovars of *Psi,* namely, *Psi* pv. *cepacicola* pv. nov. and *Psi* pv. *setariae* pv. nov. [7]. The pathovar *Psi* pv. *cepacicola* causes symptoms on the *Allium* species (leek, onion, chive and Japanese bunching onion) and also on foxtail millet, pearl millet, and oat. However, *Psi* pv. *setariae* pv. nov. can only infect the members of *Poaceae* (foxtail millet, pearl millet, and oat) [7].

There has been a huge turnaround in terms of generating genomic resources mainly driven by reduction in costs associated with next generation sequencing (NGS). As a result, several studies have been conducted to comprehensively explore features specific to each genome. The most widely explored genomic variants used in genome-wide studies are single nucleotide polymorphisms (SNPs) that are utilized in both prokaryotes [8] and eukaryotes [9,10,11,12,13,14]. Another widely used technique to explore variations in prokaryotic genomes is the use of presence and absence variants (PAVs). These PAVs capture an often evolving “accessory genome” of an organism. Another part of the genome(s), which is conserved is regarded as a “core genome”. Together, the core and the accessory genomes constitute a pan-genome of a species or sometimes across species for a given genus (also regarded as super pan-genome) [15]. Hence, the core genome refers to key genes that commonly exist in every member of a specific genome set, and the accessory genome represents dispensable genes, which only exist in some of the genomes [16]. In prokaryotes, a pan-genome can be open or closed depending on the similarity of the gene content. Genomes with highly similar gene content make a closed pan-genome and genomes with diverse gene content makes a pangenome open [17]. A pan-genome of a species is dependent on the number of genomes involved in the dataset, the ability to integrate exogenous DNA into its genome via horizontal gene transfer (HGT), and its ecological niche [18]. The gain or loss of genes in bacteria are often mediated by HGT events and are among the key processes in bacterial evolution [19]. HGT can result in the replacement of genetic segments with donor homologues, often within species via. homologous recombination, or via. the acquisition of new genetic material.

In our earlier pan-genome study, we used 81 strains of *P. ananatis* (including both pathogenic and non-pathogenic strains of onion) and performed pan-GWAS (pan-genome wide association study) to predict genes involved in onion pathogenicity [8]. Our pan-GWAS study was able to predict genes and gene clusters potentially involved in onion pathogenicity and predicted several HGT events that occurred between onion-pathogenic vs. onion-non-pathogenic strains. In addition, phylogeny based on PAVs was also able to differentiate onion-pathogenic vs. non-pathogenic strains. In the current study, we utilized the genome of *Psi* strains from both pathovars [pv. *cepacicola* (*n* = 4) and pv. *setariae* (*n* = 13)] and developed a pan-genome with a conserved core and a flexible accessory genome. Further, we performed a pan-GWAS study to identify genes in *Psi* pv. *cepacicola* that are associated with onion pathogenicity and also predicted several HGT events that occurred between *Psi* and other *Pantoea* spp. (*P. stewartii* subsp. *stewartii* LMG 2715^T^, *P. ananatis* LMG 2665^T^*, P. agglomerans* LMG L15, *P. allii* LMG 24248^T^). We further utilized SNPs and PAVs of the core-genome to assess the phylogeny of both *Psi* pathovars.

## 2. Methods

### 2.1. Bacterial Strains, Identification, and Culture Preparation

Seventeen *Psi* strains from both pathovars [pv. *cepacicola* (*n* = 4) and pv. *setariae* (*n* = 13)] (Table 1) were used in this study that were phenotypically characterized in our earlier study [7]. Out of the 17 strains, two strains, *Psi* pv. *setariae* LMG 2632^T^ (NZ_JPKO00000000.1) and *Psi* pv. *setariae* PNA 03-3 (GCA_003201175.1), were previously sequenced. Genome assemblies of the rest of the 15 *Psi* strains have been submitted to NCBI under the Bioproject ID PRJNA670043 (genome submission: SUB8606059). Whole-genome sequences of four types of strains of *Pantoea* spp. (*P. stewartii* subsp. *indologenes* pv. *setariae* LMG 2632^T^ (NZ_JPKO00000000.1); *P. stewartii* subsp. *stewartii* LMG 2715^T^ (GCA_008801695.1); *P. ananatis* LMG 2665^T^ (NZ_JMJJ00000000); *P. allii* LMG 24248^T^ (NZ_NTMH00000000)) and WGS of *P. agglomerans* L15 (NZ_CP034148) were used. Genomic features of all 21 strains used in this study are listed in Table 1.

### 2.2. Identification of Presence and Absence Variations (PAVs) and Core Genome Phylogeny

The gbk (genebank format) files of the draft-assembled and annotated genomes of *Psi* strains [7] using gethomologues [20] were used for pan-genome analyses. These gbk files were used to acquire the syntenic sequence clusters by get_homologues.pl using the OrthoMCL (OMCL) algorithm. The syntenic clusters generated were used to develop a pan-genome matrix showing the presence and absence variants (PAVs) using compare_clusters.pl. The matrix was also used to classify genes into the core, soft-core, shell and cloud genes using the parse_pangenome_matrix.pl (auxiliary script of get_homologues.pl). Core genes are defined as those present in all 17 *Psi* genomes whereas accessory genes are present in a subset of the 17 genomes. The accessory gene cluster was further divided into cloud and shell gene clusters. Soft-core genes occurred in 95% of the genomes. Cloud genes were present in ≤2 genomes and shell genes were comprised of the remaining genes [20]. Distribution of cluster sizes as a function of the number of genomes these clusters contained was displayed using R with parse_pangenome_matrix.pl. Gower’s distance matrix was generated using the tab delimited pan-genome PAV file as the input and then executing shell script hcluster_pangenome_matrix.sh (auxiliary script of get_homologues) with R function hclust. A core genome phylogeny was produced using Parsnp of Harvest suite [21], which makes an alignment from localized co-linear blocks. The alignment was run using -r!, -c and -x options where, -r! was used to select a random reference genome from the list of genomes used in analysis, -c was used to include all 17 genomes forcibly in the core genome analysis, and -x flag was used to enable filtering of SNPs located in PhiPack [22]-identified regions of recombination. All polymorphic columns in the multiple alignment were flagged to identify: (1) repetitive sequence; (2) small LCB size; (3) poor alignment quality; (4) poor base quality; and (5) possible recombination. After running all filters, columns with filtered SNPs were displayed in the image. The final set of core-genome SNPs was used as an input to FastTree2 [23] for reconstruction of the whole-genome phylogeny. Since Harvest is developed to conduct intraspecific core-genome alignment studies, we used it only on the 17 *Psi* strains and did not include other four species/subspecies of the *Pantoea* complex. Gingr [21], a dynamic visual platform of Harvest, was used for visualizing the phylogenetic tree.

### 2.3. Horizontal Gene Transfer (HGT) and Phylogenetic Analysis of Genomes of Pantoea Complex

A phylogenetic tree was built for all input genomes using the protein sequences of universal single copy genes (SCGs). To carry out HGT analysis, we included genomes of four additional strains representing the four *Pantoea* species of the *Pantoea* complex, namely, *P. stewartii* subsp. *stewartii* LMG 2715^T^, *P. ananatis* LMG 2665^T^, *P. allii* LMG 24248^T^ and *P. agglomerans* L15. Predicted protein sequences of all 21 genomes (17 *Psi* and four *Pantoea* spp. stated above; Table 1) were used to search for the HMM (PFAM and TIGRFAM) [24,25] profiles of these SCG proteins using HMMER [26]. Protein sequences for each HMM profile were aligned using HMMER and were concatenated into a single-multiple-sequence alignment using the GTDB tool kit [27]. Further, we utilized multiple-sequence alignment file to build a phylogenetic tree. The tree file was visualized in iTOL [28]. Customized groupings (A, B, C, and D) were made based on the tree. These customized groups of genomes were used as input to study HGT events among the *Pantoea* spp. mentioned above. MetaCHIP [29] was used to identify horizontal gene transfer (HGT) among the customized assigned groups. MetaCHIP identified putative donor and recipient transfer events within the 21 *Pantoea* strains (17 *Psi* and four *Pantoea* spp. stated above; Table 1) based on combined similarity and phylogenetic incongruency.

SNPs from the core genome and PAVs of *Psi* strains (*n* = 17) and other *Pantoea* spp. including *P. stewartii* subsp. *stewartii* LMG 2715^T^, *P. ananatis* LMG 2665^T^, *P. allii* LMG 24248^T^ and *P. agglomerans* L15 were identified, and a phylogenetic tree was constructed. Pan-seq pipeline [30] was used to identify the core SNPs, and PAVs from the accessory genomes. The output phylip files were used to construct phylogenetic trees based on SNPs and PAVs. The PHYLIP files were imported into RaxML software [31] and PHYLIP trees were constructed by using the “neighbor-joining” method with a bootstrap setting of 1000. Further, a whole genome multi locus sequence typing (wgMLST) tree was also constructed. To carry out this analysis, assembled contigs files of each of the 21 *Pantoea* spp. strains were uploaded to PGAdb builder and a pan-genome allele database was constructed with 1000 iterations [32].

### 2.4. Pan-Genome-Wide Association Analysis and Annotations

We utilized phenotypic data from Koirala et al. (2021) [7] where both *Psi* pv. *cepacicola* and *Psi* pv. *setariae* strains were phenotyped based on their ability to cause symptoms on onion seedlings. Only four of the *Psi* pv. *cepacicola* strains (PNA 03-3, PNA 14-9, PNA 14-11 and PNA 14-12) strains were able to cause foliar symptoms. Scoary [33] was used to calculate associations among genes in the pan-genome and the pathogenic phenotype on onion seedlings (a qualitative assessment; pathogenic vs. non-pathogenic association). The output of this program comprised of a list of genes sorted by strength of association with these traits. Genes with a naïve *p*-value ≤0.005 and corrected *p*-value (Benjamini-Hochberg) of association < 0.25 were considered significant. The core, soft-core, shell, and cloud genes were retrieved from the *P. stewartii* pan-genome and subjected to blastX against the NR database. The blast output files generated in.xml format were used as input to blast2GO. First, GO mapping was performed to retrieve the GO terms associated with blast to form a pool of GO terms. Then GO annotation was carried out where the GO terms from the pool of GO terms were assigned to query sequences. All sequences with GO annotations were also annotated for enzyme code. GO term associations were classified and plotted as biological process (BP), molecular function (MF), and cellular component (CC). The FatiGO [34] package integrated into Blast2GO was used for statistical assessment of annotation differences as following: core vs accessory, soft-core vs core, shell vs core, and cloud vs core genes. This package uses the Fisher’s Exact Test and corrects for multiple testing. Adjusted *p*-values of each GO term were reported based on the corrected *p*-value by False Discovery Rate (FDR) control. Genes involved in horizontal gene transfer (HGT) were annotated using Blast2GO pipeline [35].

## 3. Results

### 3.1. The P. stewartii subsp. indologenes Pan-Genome Architecture and Phylogeny

**Figure 1:**
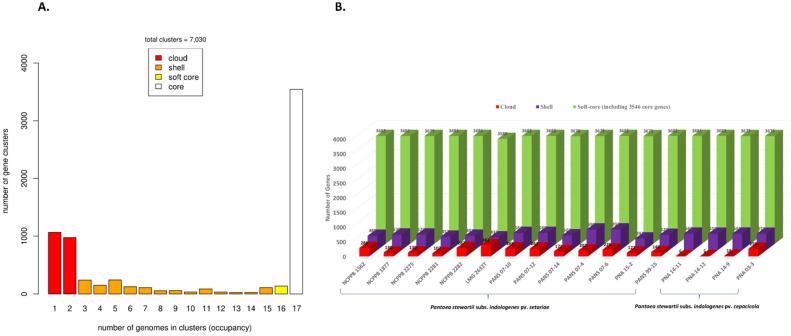
Pan-genome analysis of 17 *Pantoea stewartii* subsp. *indologenes* genomes. (**A**) Distribution of gene (cluster) sizes as a function of the number of genomes; (**B**) Genes contributed by each genome that contributed to the pan-genome.

Genome assembly is a pre-requisite to study the pan-genome of a given species. Assembly sizes of the seventeen strains of *Psi* ranged from 4.6 (PNA 15-2) to 5.1 Mbp (PANS 07-6) with the number of contigs ranging from 22 (PNA 03-3) to 125 (PANS 07-4) and the number of genes ranging from 4341 (PNA 15-2) to 4816 (PANS 07-6) (Table 1). The full spectrum of the pan-genome of *Psi* contained 7030 genes. Among these, 3546 genes (present in all 17 strains) were part of the core genes and were a part of a subset of 3682 soft-core genes (present in ≥16 strains). The accessory genome included 1308 shell genes and 2040 cloud genes (present in ≤2 strains) (Figure 1A). The genome of each strain contributed a conserved set of 3546 core genes and a variable number of accessory genes. Overall, soft-core genes contributed by each genome ranged from 3580 to 3682 genes, including a conserved set of 3546 genes. Shell genes ranged from 394 to 703 genes and the cloud genes ranged from a minimum of five genes contributed by *Psi* pv. *cepacicola* PNA 14-12 to a maximum of 382 genes contributed by *Psi* pv. *setariae* LMG 2632^T^ (Figure 1B). Details of the number of core and accessory genes contributed by each strain are listed in (Table S1). 

**Figure 2:**
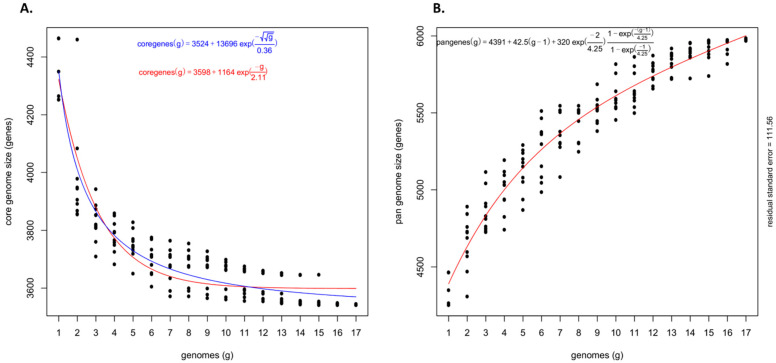
Theoretical estimation of the core and pan-genome sizes based on the exponential decay model. (**A**) Estimation of core genome size based on the Willenbrock model fit to OMCL clusters and (**B**) Estimation of pan-genome size based on the Tettelin model fit to OMCL clusters.

The pan-genome architecture of the 17 *Psi* genomes, which included both pathovars, revealed an open pan-genome (Figure 2). Exponential decay models [16,36] that fitted the core gene clusters predicted a theoretical core genome of 3598 and 3524 genes (Figure 2A). Further, to confirm the openness/closeness of the pan-genome, the theoretical estimation of the pan-genome size was carried out using Tettelin’s exponential model fitted to the OMCL accessory gene clusters. The pan-genome showed a clear linear progression with >6000 genes, with ~43 new genes added on an average to the pan-genome as each new *Psi* genome was added (Figure 2B). This indicated an open *Psi* pan-genome.

**Figure 3:**
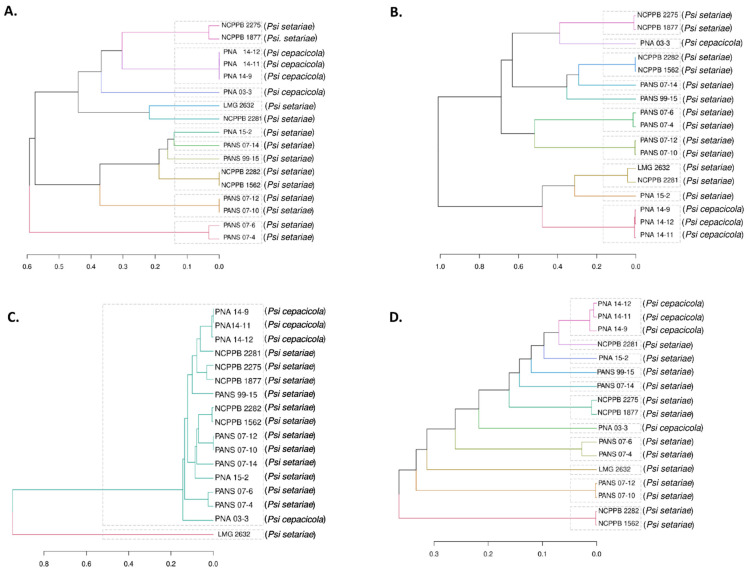
Dendrogram of 17 strains of *Pantoea stewartii* subsp. *indologenes* based on the core and accessory genes. (**A**) Dendrogram based on core genes, i.e., genes present in all 17 strains used in the study; (**B**) Dendrogram based on soft-core genes, i.e., genes present in at least 95% of the strains; (**C**) Dendrogram based on shell genes; and (**D**) Dendrogram based on cloud genes, i.e., the genes specific to each strain or shared by a maximum of two strains.

Dendrograms were plotted based on the core and accessory genes identified in the *Psi* pan-genome. The four components of the developed pan-genome of *Psi,* namely, core, soft core, shell, and cloud genes, were used to assess the phylogeny of 17 *Psi* strains. All four components of the pan-genome used individually clustered the *Psi* pv. *cepacicola* strains separately from the *Psi* pv. *setariae* strains. However, only one *Psi* pv. *cepacicola* (PNA 03-3) was distantly clustered with *Psi* pv. *setariae* strains when soft-core, shell, and cloud genes were used. The *Psi* pv. *cepacicola* strain PNA 03-3 clustered close to other three *Psi* pv. *cepacicola* strains (PNA 14-12, PNA 14-11, PNA 14-9) only when core genes were used (Figure 3A–D).

### 3.2. Core Genome Genes Differentiated the Onion-Pathogenic P. stewartii subsp. indologenes pv. cepacicola Strains from the Onion-Non-Pathogenic P. stewartii subsp. indologenes pv. setariae Strains

Among the seventeen newly discovered *Psi* strains, four *Psi* pv. *cepacicola* strains were pathogenic on *Allium* spp. (onion, leek, chive, and bunching onion) and *Poacea* (oat, rye, foxtail millet) species [7]. The remaining strains belonged to *Psi* pv. *setariae*, and were pathogenic only on *Poacea* species (oat, rye, foxtail millet). Clustering based on the ANI matrix of pan-genome resulted in phylogenic trees based on core, soft-core, shell, and cloud genes (Figure 3). Core genes ANI matrix resulted in eleven clusters with three of the four *Psi* pv. *cepacicola* strains clustered together and the fourth strain (PNA 03-3) placed separately (Figure 3A). Soft-core ANI resulted in ten clusters (Figure 3B). Shell and cloud genes (accessory genome) ANI resulted in two and eleven clusters, respectively. Two shell gene clusters contained 16 out of the 17 *Psi* strains in one cluster and one strain (LMG 2632^T^) in a separate clade (Figure 3C). Three out of the four *Psi* pv. *cepacicola* strains (PNA 14-9, PNA 14-11, and PNA 14-12) consistently clustered together with core or accessory genes-based ANI.

### 3.3. Horizontal Gene Transfer (HGT) and Annotation of Genes Involved in HGT

**Figure 4:**
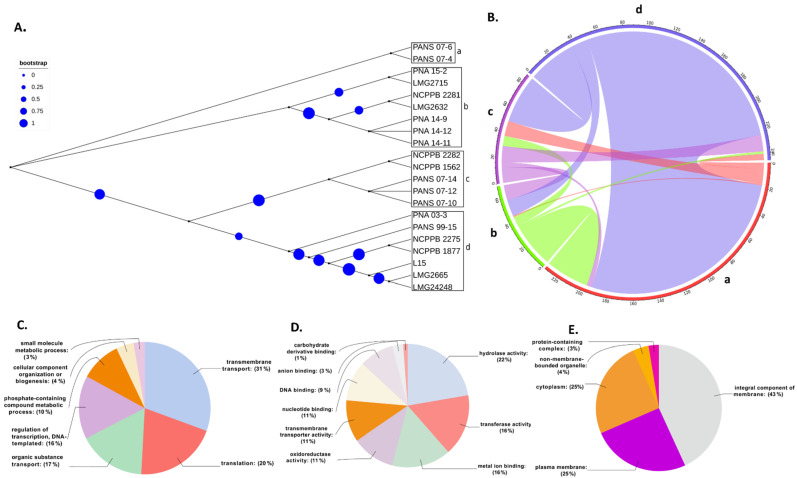
Phylogeny-based horizontal gene transfer (HGT) among 17 strains of *Pantoea stewartii* subsp. *indologenes* strains and four other species of the *Pantoea* complex, namely, *P. ananatis*, *P. stewartii* subsp. *stewartii*, *P. agglomerans,* and *P. allii*. (**A**) Phylogenetic tree of *Pantoea* spp. (*n* = 21) strains based on multiple-sequence alignment. Phylogenetic tree resulted in four clusters (a–d). Size of circles represent the bootstrap values in that order; (**B**) Predicted gene flow within the four phylogenetic clusters of *Pantoea* spp. Bands connect donors and recipients, with the width of the band correlating to the number of HGTs and the color corresponding to the donors. Numbers on the circumference of circos plot represent the number of genes that underwent horizontal gene transfers. The four arcs (a–d) of circos represent the four phylogenetic clusters of *Pantoea* spp. strains; (**C**–**E**) Graphical annotations of sequences involved in HGT as per the assigned GO terms: (**C**) shows the function of genes assigned to biological processes, (**D**) shows the function of genes assigned to molecular functions, and (**E**) represents the function of genes assigned to cellular components.

Five *Pantoea* species including 17 *Psi* strains (4 *Psi* pv. *cepacicola* and 13 *Psi* pv. *setariae* strains) and one strain each of *P. ananatis* (LMG 2665^T^), *P. allii* (LMG 24248^T^), *P. agglomerans* (L15), and *P. stewartii* subsp. *stewartii* (LMG 2715^T^) were used for HGT analysis. Phylogenetic classification based on the conserved SCG resulted in four groups (a–d) (Figure 4A). Strains classified within these four groups were used to study the HGT as explained in the Section 2. A total of 317 HGT events including 314 donor and 299 recipient genes among the five *Pantoea* species/pathovars were identified (Figure 4B, Table S2). Most of the gene transfers (*n* = 95) occurred from PNA 03-3 (*Psi* pv. *cepacicola*) to PANS 07-4 (*Psi* pv. *setariae*), followed by 76 HGTs from NCPPB 2275 (*Psi* pv. *setariae*) to PANS 07-4 (*Psi* pv. *setariae*), 32 from NCPPB2281 (*Psi* pv. *setariae*) to PANS 07-4 (*Psi* pv. *setariae*), and 27 from NCPPB 2275 to PNA 07-10 (*Psi* pv. *setariae*). The strain NCPPB 2275 (*Psi* pv. *setariae*) transferred (donated) a maximum number of 112 genes, followed by PNA 03-3 (*Psi* pv. *cepacicola*; *n* = 100) and NCPPB 2281 (*Psi* pv. *setariae*; *n* = 35), and others donated less than 20 genes. Similarly, among the recipient strains, PANS 07-4 (*Psi* pv. *setariae*) received a maximum of 211 genes followed by PANS 07-10 (*Psi* pv. *setariae*; *n* = 40) and the remaining strains received less than 20 genes (Figure 4B, Table S2). There were two compulsive donor strains (*Psi* pv. *setariae* NCPPB 2275 and *Psi* pv. *setariae* NCPPB 2281) that did not feature as recipients. Similarly, two recipient strains (*Psi* pv. *setariae* LMG 2632^T^ and *Psi* pv. *setariae* NCPPB 1562) did not feature as donors (Figure 4B, Table S2).

### 3.4. Annotations of Genes Involved in Horizontal Gene Transfer (HGT)

**Figure 5:**
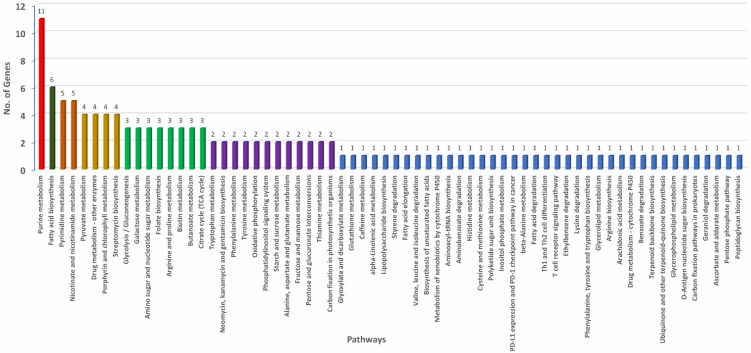
Annotated genes involved in horizontal gene transfer (HGT) with functions assigned under biochemical, metabolic, and physiological pathways operational in *Pantoea stewartii* subsp. *indologenes*.

Further, the donor and recipient proteins coded by genes involved in HGT were annotated. A non-redundant set of 607 genes coding for proteins involved in HGT showed blast hits. A total of 499 out of 607 genes that showed blast hits were mapped and annotated (Table S3). Each of the 499 genes involved in HGT were assigned GO IDs from a minimum of one to a maximum of eight. Eight genes were assigned to a maximum of eight GO IDs followed by 23 genes assigned to seven, 26 genes to six, 57 genes to five, 86 genes to four, 107 to three, 80 genes to two, and the remaining 112 genes to one GO ID (Table S4). Among all the HGTs, ABC transporter permease featured in a maximum of eight HGT events followed by GNAT family N-acetyltransferase in seven, cytochrome ubiquinol oxidase subunit I in six, and outer membrane lipoprotein chaperone in five HGT events (Table S3). Based on the assigned GO IDs, HGT genes were classified into biological process (BP), molecular function (MF), and cellular component (CC). Under BP, the maximum number of genes involved in HGT were involved in transmembrane transport (31%), followed by genes involved in translation (20%), and only 3% were involved in small molecule metabolic processes (Figure 4C). Under MF, 22% were categorized with hydrolase activity, followed by 16% that were categorized as transferase activity and metal binding. The smallest number of HGTs were categorized as carbohydrate-derivative binding (Figure 4D). In CC, most of the genes were categorized under integral component of membrane (43%) followed by 25% each under plasma membrane and cytoplasm and the minimum number as a protein-containing complex (3%) (Figure 4E). Further, we investigated various pathways that these donor and recipient genes were involved. A total of 66 biochemical pathways were identified using the KEGG database where HGT genes were involved. Purine fatty acid biosynthesis, pyrimidine, nicotinate, and nicotinamide metabolic pathways featured most of the HGT genes. Purine metabolism showed 11 genes, fatty acid biosynthesis showed six, and pyrimidine and nicotinamide metabolism showed five genes each. Remaining metabolic pathways involved three, two, or one gene(s) (Figure 5, Table S5).

### 3.5. Comparative PAVs, Core SNPs and Whole Genome Multi Locus Sequence Typing (wgMLST) Based Phylogeny

**Figure 6:**
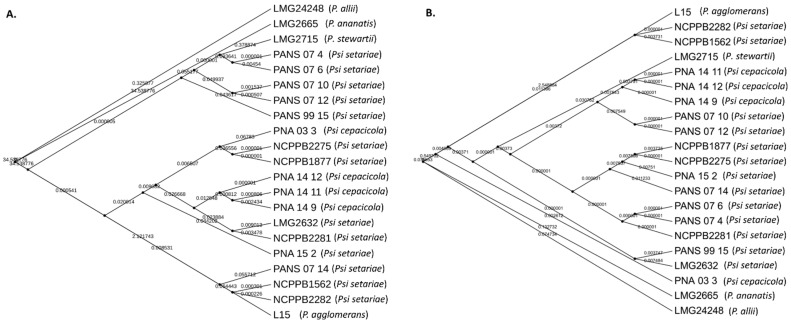
Comparative phylogeny of onion-pathogenic and onion-non-pathogenic strains of *Pantoea stewartii* subsp. *indologenes* based on core genome SNPs and presence and absence variations (PAVs). (**A**) Phylogenetic tree constructed using PAVs using RAxML; (**B**) Phylogenetic tree constructed using core SNPs using RAxML. Numerical values in decimal represent the branch length. Longer branch length indicates higher genetic divergence.

**Figure 7:**
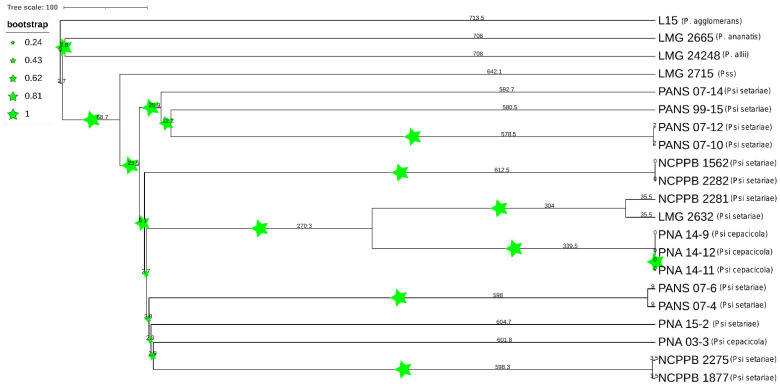
Phylogenetic tree based on whole genome multi-locus sequence typing (WgMLST). Dendrogram for 21 *Pantoea* spp. strains was constructed using assembled genome contigs. Size of stars represents the boot strap values and numbers represent the branch length. PSS is *Pantoea stewartii* subsp. *stewartii*.

A comparative phylogenetic analysis was conducted using PAVs and core genome SNPs. Phylogenomic analysis using PAVs and SNPs showed both *P. ananatis* (LMG 2665^T^) and *P. allii* (LMG 24248^T^) were outliers. However, *P. agglomerans* (L15) grouped together with the same *Psi* pv. *setariae* strains using both PAVs and SNPs (Figure 6). The strain *P. stewartii* subsp. *stewartii* (LMG 2715^T^) clustered together with PANS 07-4 (*Psi* pv. *setariae*) and PANS 07-6 (*Psi* pv. *setariae*) when plotted using PAVs. However, core SNPs were used and clustered with *Psi* pv. *cepacicola,* although they did not share the same node. Three of the four *Psi* pv. *cepacicola* strains (PNA 14-12, PNA 14-11, and PNA 14-9) clustered together when both with PAVs and core SNPs were used. The fourth *Psi* pv. *cepacicola* strain (PNA 03-3) clustered with *Psi* pv. *setariae* strains when PAVs were used. However, when core SNPs were used, PNA 03-3 formed a separate clade between LMG 2632^T^ (*Psi* pv. *setariae*) and LMG 2665^T^ (*P. ananatis*). Overall, with both PAVs and SNPs the *Psi* pv. *cepacicola* and *Psi* pv. *setariae* strains clustered in two separate groups, except for PNA 03-3 (Figure 6). PAVs along with SNPs were identified using pan-seq to conduct comparative phylogenetic analysis. As seen with SNPs and PAVs, wgMLST clustered *Psi* pv. *cepacicola* strains together except for the strain PNA 03-3, which clustered with *Psi* pv. *setariae* strains (Figure 7). Strains of four other species of *Pantoea* used in this study (*P. agglomerans*, *P. ananatis*, *P. allii*, *P. stewartii* subsp. *stewartii*) branched separately from *Psi* strains used. Like the PAV-based phylogenetic tree, the wgMLST tree showed *P. stewartii* subsp. *stewartii* (LMG 2715^T^) to be the closest to *Psi* pv. *setariae* out of the four Pantoea spp. compared (Figure 6 and Figure 7). However, wgMLST-based phylogeny branched out four *Pantoea* species from *Psi* strains, which was not observed in PAV or SNP-based phylogeny. Further, to rule out the possibility that recombination events within the 17 *Psi* strains could interfere with core-genome-SNP-based phylogeny, we conducted a phylogenetic study of *Psi* strains where recombination sites were excluded (Figure S1). The results showed that the *Psi* pv. *cepacicola* strain (PNA 03-3) clustered away from the remaining three *Psi* pv. *cepacicola* strains, as was observed with PAVs-, SNPs- and wgMLST-based phylogeny.

### 3.6. Pan-Genome-Wide Association Study

An association study was conducted using the qualitative phenotyping data (pathogenicity on onion seedling) and the core and accessory genes. The goal was to identify genes responsible for pathogenicity of *Psi* strains in onion. Scoary predicted a total of 154 genes associated with the phenotype (Table S6). Among the 154 genes, we found seven clusters of genes associated with the phenotype. There were three gene clusters with 5, 12, and 11 genes that were highly significant based on *p*-values (*p*-value ≤ 0.005). Two out of the three clusters contained only non-annotated hypothetical protein coding genes. However, one cluster contained 11 protein coding genes (Table 2). These 11 genes coded for Pyridoxal 5’-phosphate synthase, AMP-binding protein, MFS transporter, phosphoglycerate kinase, FAD-NAD(P)-binding protein, phosphoenolpyruvate phosphomutase, NAD(P)-binding domain-containing protein, N-acetyl-gamma-glutamyl-phosphate reductase, alcohol dehydrogenase catalytic domain-containing protein, iron containing alcohol dehydrogenase, and a LysE-family-translocator. A Blast search against the PNA 97-1R genome assembly (GCA_002952035.2) identified all genes except the last gene (LysE-family translocator) in the cluster. Interestingly, the first 10 genes in the cluster are chromosomally localized, but the last gene is localized in the large plasmid (Table S7).

### 3.7. Annotation of Pantoea stewartii subsp. indologenes Pan-Genome

The core and accessory genomes were annotated as genes involved in BP, MF, and CC (Figure S2). Under BP: response to stimulus, metabolic process, and cellular process were common in core, soft-core, shell and cloud genes. Regulation of biological process was specific to soft-core genes and shell genes lacked biological regulation and regulation of biological process functions. Under MF, catalytic and binding activities were common in all the core and accessory genes. However, genes coding for molecular function regulators were specific to the soft-core component of the pan-genome and shell genes lacked transporter activity. The genes in the CC category were assigned to the cellular anatomical entity and intracellular component (Figure S2). Further, we performed statistical assessment of annotation differences between core and accessory genes. When the core genome was compared with the accessory genome, catalytic activity, cellular process, metabolic (cellular) process, binding and nitrogen compound metabolic processes were the top six highly represented GO terms. When the soft-core was compared with the core, organic cyclic/heterocyclic compound binding, intrinsic/integral membrane component, small molecule binding, and localization were highly represented GO terms. When shell genes were compared with core-genes, they showed nucleic acid binding, heterocyclic/organic cyclic compound binding, DNA binding, and macromolecule (cellular) metabolic process as highly represented GO terms. Similarly, cloud genes compared with core-genes showed the same highly represented GO terms. However, cloud vs. core was different from shell vs. cloud in terms of intracellular (membrane) bounded organelle, extracellular space, and multicellular-organismal process GO terms specific to the cloud genome. Cytoskeleton organization and symbiotic process were specific to shell only (Figure S3).

## 4. Discussion

*Pantoea stewartii* subsp. *indologenes* causes a leafspot of foxtail millet and pearl millet, a rot of pineapple, and one strain has also been isolated from the cluster bean (*Cyamopsis tetragonolobus*) [6]. Recently, *P. stewartii* subsp. *indologenes* strains (*Psi* pv. *cepacicola*) were identified that caused symptoms similar to the center rot of onions [2]. These bacterial species/pathovar are not as prevalent as *P. ananatis* in Georgia or elsewhere but are known to cause center rot disease in onions. Genome analysis provides insights about the genes involved in pathogenicity and probable virulence mechanism(s). Moreover, it is important to know the architecture of pan-genome, phylogeny, and evolutionary association of *P. stewartii* subsp. *indologenes* strains with other *Pantoea* spp. and similarity or uniqueness in the onion-virulence repertoire. We therefore conducted a pan-genome study of seventeen newly identified *Psi* strains from both pathovars [pv. *cepacicola* (*n* = 4) and pv. *setariae* (*n* = 13)] and developed a core and a pan-genome followed by annotation of the core and accessory genes. Further, we carried out a pan-GWAS study and identified gene(s) associated with pathogenicity in onions. Phylogenetic and HGT studies were also conducted to understand the role that SNPs, PAVs, and HGTs have on the phylogeny of these strains.

### 4.1. Pantoea stewartii subsp. indologenes Pan-Genome and Horizontal Gene Transfer

In the current study, we identified 3546 core genes, 5 to 382 cloud genes, and 394 to 703 shell genes. A similar study using 81 *P. ananatis* strains identified 3153 core genes and cloud genes ranging from 1000 to 6808 [8]. We observed a stark difference in the number of cloud genes when compared with our earlier study on *P. ananatis*. A slightly higher number of core genes and a lesser number of cloud genes identified in the current study are expected to change in the future with an increase in the number of *Psi* genomes being added to the pan-genome. Fewer accessory genes were observed as compared with our previous study [8]. Fewer accessory genes in this study can be a result of HGT events because gene exchanges due to HGT can lead to extensive gene repertoire differences among closely related species or within species [37]. This may partly explain why a smaller number of cloud genes were identified in three *Psi* pv. *cepacicola* strains (PNA 14-11, PNA 14-12, and PNA 14-9) as compared with the rest of the *Psi* strains used in this study. Perhaps as more *Psi* pv. *cepacicola* genomes are included, the number of core genes may decrease and accessory genes may increase, as indicated in other bacterial pan-genomes (*Escherichia coli*) [38].

A dynamic pan-genome is also dependent on the frequent HGT events it encounters during evolution. We therefore studied HGT within *Psi* strains and among the four species of the *Pantoea* complex (*P. ananatis*, *P. agglomerans, P. stewartii* subsp. *stewartii,* and *P. allii*). The maximum number of HGT events occurred from PNA 03-3 (*Psi* pv. *cepacicola*) to PANS 07-4 (*Psi* pv. *setariae*), which may explain the phylogenetic clustering of PNA 03-3 with PANS 07-6 and PANS 07-4 using the shell and cloud genes. This in contrast to three *Psi* pv. *cepacicola* strains, namely, PNA 14-11, PNA 14-12, and 14-9 that contained 6, 5, and 18 cloud genes, respectively; *Psi* pv. *cepacicola* strain PNA 03-3 contained 259 cloud genes, which is similar to the number of cloud genes exhibited by *Psi* pv. *setariae* strains (range: 107 to 382). It seems that HGT events deeply impacted the gene loss in the abovementioned three *Psi* pv. *cepacicola* strains.

### 4.2. An Open Pan-Genome of Pantoea stewartii subsp. indologenes

The pan-genome approach is important for exploring the genomic repertoires of a phylogenetic lineage of microbes [39]. The pan-genome of *Psi* showed a linear progression with >6000 genes, with ~43 new genes adding on average to the pan-genome with each new *Psi* genome sequenced, suggesting that it was open and attributed to frequent evolutionary changes mediated by gene(s) gain and loss resulting due to HGT events. The open feature of *Psi* was consistent with *P. ananatis* and *Geobacillus* spp. datasets [8,17,40], as opposed to other species, such as *Bifidobacterium breve* and *Staphylococcus lugdunensis*, which depicted a closed trend [41,42].

### 4.3. Phylogenetic Study of Pantoea stewartii subsp. indologenes and Other Pantoea spp. Complex

A comparative phylogenetic approach was undertaken to evaluate the phylogeny of both *Psi* pathovars compared to other *Pantoea* spp. We used PAVs, SNPs, and wgMLST approaches to understand the differences in the phylogeny of *Psi* strains along with four *Pantoea* spp. based on the underlying genomic variants. The core genome with conserved genes instead of accessory genome with PAVs may convey a true measure of phylogeny. We therefore inferred our phylogenetic analysis based on core genome analysis. We found ANI based on core genes clustered *Psi* pv. *cepacicola* strains closely. However, soft-core genes ANI clustered one *Psi* pv. *cepacicola* strain (PNA 03-3) distantly from the other three strains. Similar, observations were made when shell and cloud genes were used to conduct the phylogenetic analysis. This suggests that onion-pathogenic *Psi* pv. *cepacicola* strains and onion-non-pathogenic *Psi* pv. *setariae* strains could be distinguished using core genes, which was not the case with accessory genes. Clustering based on the core and accessory genome ANI showed a slight difference, indicating the evolutionary and pathogenicity relationship was better depicted with core genes than the accessory genes. However, the number of input genomes may have a role to play and core genes-based phylogeny could probably change if the number of strains/genomes is further increased. It could also be possible that the core genome is impacted by HGT or homologous recombination and, as a result, the phylogenetic relationship based on core genes is obscured or distorted [43].

SNPs are vertically inherited and are one of the dominant forms of evolutionary change that have become an indispensable tool for phylogenetic analyses [44,45,46]. Hence, core genes were used to classify onion pathogenic *Psi* pv. *cepacicola* vs. onion non-pathogenic *Psi* pv. *setariae* strains. This was performed by identifying SNPs from the core genome and performing a phylogenetic analysis. Core SNPs clustered *Psi* pv. *cepacicola* strains together except for one strain, PNA 03-3.

Genomic regions that may have been involved in past recombination events should be excluded from phylogenetic analyses to produce more accurate phylogeny [47]. Therefore, we conducted another core-genome-SNPs-based phylogeny wherein the recombination events were excluded; however, despite the removal, we found similar results that we observed using SNPs- and PAVs-based phylogeny. We expected that excluding the recombination sites would result in clustering of PNA 03-3 (*Psi* pv. *cepacicola*) with the other three *Psi* pv. *cepacicola* strains (PNA 14-11, PNA 14-12 and PNA 14-9); however, that was not the case. This may in part indicate that the strain PNA 03-3 is evolutionarily distinct from the other *Psi* pv. *cepacicola* strains (PNA 14-11, PNA 14-12, and PNA 14-9).

Our earlier study showed that *Psi* pv. *cepacicola* PNA 03-3 was highly aggressive on leek, foxtail millet, and pearl millet, whereas it was moderately aggressive on onion, chive, Japanese bunching onion, and oat. Other *Psi* pv. *cepacicola* strains (PNA 14-9, PNA 14-11, PNA 14-12) were moderately-to-highly aggressive on onion, foxtail millet, and pearl millet but were moderate-to-less aggressive on leek, chive, Japanese bunching onion, and oat [7]. We believe that this difference in aggressiveness is represented by core SNPs. Additionally, *Psi* pv. *cepacicola* PNA 03-3 is more closely related to *P. ananatis* (LMG 2665^T^), which is reflected in the core SNPs-based phylogeny. The WgMLST, an extended concept of MLST, is complementary to PAVs-based phylogenetic analysis [8]. As expected, we observed a similar pattern of phylogenetic classification of strains both with PAVs- and wgMLST-based phylogeny. Particularly, *Psi* pv. *cepacicola* PNA 03-3 was clustered closely with *Psi* pv. *setariae* strains (NCPPB 2275 and NCPPB 1877) in both PAVs- and wgMLST-based phylogenetic analysis. These observations reiterated potential phylogenetic and evolutionary differences between the *Psi* pv. *cepacicola* strains, particularly with PNA 03-3 vs. PNA 14-11, PNA 14-12, and PNA 14-9.

### 4.4. Gene Cluster Identified from Pan-GWAS Analysis

The PAVs identified using *Psi* strains (*n* = 17) and the phenotyping data, when subjected to pan-GWAS, identified a gene cluster associated with pathogenicity in the *Allium* species. The phenotyping data were utilized from our earlier study [7]. Pan-GWAS identified several genes that were associated with the pathogenicity on onion seedlings. Out of all the genes identified (*n* = 154), a cluster of 11 well-annotated genes was identified and found to be strongly associated with pathogenicity in onion seedlings. Ten genes out of the eleven associated genes were found to be present in three out of the four pathogenic *Psi* pv. *cepacicola* strains (PNA 14-9, PNA 14-11, and PNA 14-12). However, there was one gene annotated as phosphoenolpuruvate mutase (*pepM*) that was present in all four pathogenic strains. Interestingly, based on further analysis it was found that the *pepM* gene in PNA 03-3 belongs to the previously known ‘HiVir’ cluster similar to that reported in the onion-pathogenic *P. ananatis* strains. In contrast, *pepM* gene in other *Psi* pv. *cepacicola* strains (PNA 14-9, PNA 14-11, and PNA 14-12) is a part of a novel cluster distinct from the ‘HiVir’ cluster. Hence, it is important to characterize the role of the *pepM* gene in *Psi* on onion pathogenicity in both clusters.

The novel gene cluster starts with a pyridoxal 5’-phosphate synthase (*pdxH_2*) gene, which catalyzes pentose and triose isomerizations, imine formation, amine addition, and ring formation, all in a single enzymatic system [48,49]. It is involved in ammonia transport [50]. The second gene of the cluster codes for the AMP binding protein. AMP binding proteins in bacteria are regarded as global activator proteins that are required to regulate the gene transcription [51]. The third gene (*ydeE*) in the cluster was an efflux MFS transporter known to export peptides [52]. A *Pgk-tpi* protein coding gene that is involved in the sub-pathway, which synthesizes D-glyceraldehyde 3-phosphate from glycerone phosphate (a part of the pathway glycolysis which is itself part of carbohydrate degradation), was next in the cluster. *Pgk-tpi* codes for enzymes phosphoglycerate kinase and triosephosphate isomerase that form a covalent bifunctional enzyme complex [53]. The next gene in the gene cluster codes for a FAD/NAD(P)-binding protein. The NAD(P)-binding enzymes are involved in catalyzing redox or non-redox reactions [54]. Interestingly, the next gene in the cluster (*pepM*) codes for phosphoenolpuruvate mutase, which was the first gene of the HiVir cluster identified in *P. ananatis* [55]. It was identified as the first pathogenicity factor associated with the fitness of *P. ananatis* as well as with symptom development in infected onion leaves and bulbs [55]. Potentially, the *pepM* gene (novel gene-cluster) in *Psi* pv. *cepacicola* strains (PNA 14-9, PNA 14-11, and PNA 14-12) may also play a similar role in onion-pathogenicity. As mentioned above, the role of this gene on onion-pathogenicity needs to be investigated. 

The next gene in the cluster codes for NAD(P)-binding domain-containing protein, which catalyzes the NADPH-dependent reactions. For example, hydropxypyruvate reductase carries out the reduction of glyoxylate and hydroxypyruvate into glycolate and glycerate, respectively [56,57,58]. N-acetyl-gamma-glutamyl-phosphate reductase is coded by *argC1* gene in the cluster. It catalyzes the NADPH-dependent reduction of N-acetyl-5-glutamyl phosphate to yield N-acetyl-L-glutamate 5-semialdehyde. This enzyme is involved in step 3 of the sub-pathway that synthesizes N(2)-acetyl-L-ornithine from L-glutamate. This sub-pathway itself is part of the L-arginine biosynthesis pathway [59]. Another gene in the cluster was annotated as alcohol dehydrogenase catalytic domain-containing protein. Alcohol dehydrogenases are the oxidoreductases that catalyze the reversible oxidation of alcohols to aldehydes or ketones, with the concomitant reduction of NAD^+^ or NADP^+^ [60]. The second-to-last gene in the cluster codes for another alcohol dehydrogenase, i.e., iron containing alcohol dehydrogenase (FeADH). The FeADH family has been characterized as exhibiting different catalytic activities. ADHs are capable of catalyzing a wide variety of substrates (e.g., normal and branched-chain aliphatic and aromatic alcohols, both primary and secondary alcohols, corresponding aldehydes and ketones, and polyols) and they are involved in an astonishingly wide range of metabolic processes; they are, for instance, involved in alcohol, alkane, sugar, and lipid metabolism, as well as in cell defense towards exogenous alcohols and aldehydes [61,62,63,64,65,66]. The last gene in the cluster codes for the Lyse-family translocator. The physiological function of the exporter is to excrete excess L-Lysine and L-arginine as a result of natural flux imbalances or peptide hydrolysis. It also plays important roles in ionic homeostasis, cell envelope assembly, and protection from excessive cytoplasmic heavy metal/metabolite concentrations. [67,68]. Overall, only two out of the eleven genes identified in this cluster that were common with the HiVir cluster were identified in *P. ananatis* [69]. These two common genes code for the phosphoenolpyruvate phospomutase (*PepM*) enzyme and the MFS transporter protein. These findings suggest a potential alternate set of onion pathogenicity-related genes in *Psi*, which is distinct from the known onion pathogenicity (HiVir) and virulence (allicin tolerance; *alt*) factors identified in *P. ananatis* [55,69,70]. We are currently trying to understand the role of this gene cluster in *Psi* pv. *cepacicola* on onion pathogenicity using traditional gene mutation studies.

## 5. Conclusions

The pan-GWAS approach predicted the genes associated with onion-pathogenicity in *Psi* strains, particularly in *Psi* pv. *cepacicola*. We found a cluster of genes different from the HiVir/PASVIL (identified in *P. ananatis*) cluster linked to onion pathogenicity in *Psi* pv. *cepacicola*. We concluded that there might be several pathogenicity factors involved in onion pathogenicity and to some extent these might be specific to some *Pantoea* spp. We also observed a large repertoire of accessory genes in *Psi* strains, which is suggestive of the potential for a broad and diverse niche-adaptation and host-range expansion capabilities. We observed HGT events as a major contributing factor for PAVs resulting in diversification of *Psi* and other *Pantoea* species. In the future, it would be interesting to assess if the aggressiveness of *Psi* strains on *Allium* and Poacea species can be predicted using GWAS utilizing SNPs rather than PAVs. We expect that the SNPs-based GWAS study will not only corroborate our current findings, but may potentially lead to the development of SNPs-based PCR markers that can distinguish the *Psi* pv. *cepacicola* and *Psi* pv. *setariae* strains.

## Figures and Tables

**Table 1:** Genome architecture details of *Pantoea stewartii* subsp. *indologenes* (pv. *cepacicola* and pv. *setariae*) and other *Pantoea* species used in this study.

Strain Name	*Pantoea* spp.	Biosample Accession	Genome Accession	Size (Mbp)	Contigs	CDSs	Genes	tRNAs
L15 ^$^	*P. agglomerans*	SAMN07109613	GCA_003860325.1	4.85	4	4456	4538	81
LMG 24248 ^T$^	*P. allii*	SAMN07625522	NZ_NTMH00000000	5.24	57	4855	4925	69
LMG 2632 ^T$^	*Psi setariae*	SAMN02905159	NZ_JPKO00000000.1	4.68	35	4455	4521	65
LMG 2665 ^T$^	*P. ananatis*	SAMN02740635	NZ_JMJJ00000000	4.93	17	4560	4632	71
LMG 2715 ^T$^	*P. stewartii stewartii*	SAMN12697580	GCA_008801695.1	4.52	1	4603	4677	73
NCPPB 1562 *	*Psi setariae*	SAMN16866628	JADWWO000000000	4.87	96	4524	4602	77
NCPPB 1877 *	*Psi setariae*	SAMN16866626	JADWWM000000000	4.77	83	4410	4487	76
NCPPB 2275 *	*Psi setariae*	SAMN16866625	JADWWL000000000	4.77	79	4406	4481	74
NCPPB 2281 *	*Psi setariae*	SAMN16866629	JADWWP000000000	4.70	103	4323	4399	75
NCPPB 2282 *	*Psi setariae*	SAMN16866627	JADWWN000000000	4.87	101	4529	4608	78
PANS_07_10 *	*Psi setariae*	SAMN16866621	JADWWH000000000	4.95	102	4603	4678	74
PANS_07_12 *	*Psi setariae*	SAMN16866622	JADWWI000000000	4.95	86	4602	4674	71
PANS_07_14 *	*Psi setariae*	SAMN16866623	JADWWJ000000000	4.78	90	4429	4404	74
PANS_07_4 *	*Psi setariae*	SAMN16866619	JADWWF000000000	5.05	125	4686	4760	73
PANS_07_6 *	*Psi setariae*	SAMN16866620	JADWWG000000000	5.10	117	4744	4816	71
PANS_99_15 *	*Psi setariae*	SAMN16866624	JADWWK000000000	4.81	97	4425	4498	72
PNA_15_2 *	*Psi setariae*	SAMN16866618	JADWWE000000000	4.66	92	4266	4341	74
PNA_03_3 *	*Psi cepacicola*	SAMN08776223	GCA_003201175.1	4.93	22	4571	4641	69
PNA_14_11 *	*Psi cepacicola*	SAMN16866616	JADWWC000000000	4.68	77	4317	4390	72
PNA_14_12 ^T^*	*Psi cepacicola*	SAMN16866617	JADWWD000000000	4.68	73	4307	4380	72
PNA_14_9 *	*Psi cepacicola*	SAMN16866615	JADWWB000000000	4.69	92	4325	4400	74

^$^ Sequences were downloaded from the NCBI. ^T^ Denotes type strains. * Sequences utilized from Koirala et al., 2021 study.

**Table 2:** List of genes with annotated function in the cluster identified in *Pantoea stewartii* subsp. *indologenes* by Pan-GWAS analysis.

Gene ID	Function *	Sensitivity	Specificity	Naive_*p*
58220_*pdxH_2*	Pyridoxal 5’-phosphate synthase	75	100	0.0059
58221_*dltA*	AMP-binding protein	75	100	0.0059
58222_*ydeE*	MFS transporter	75	100	0.0059
58223_*pgk-tpi*	Phosphoglycerate kinase	75	100	0.0059
58225_*spuC*	FAD/NAD(P)-binding protein	75	100	0.0059
58226_*pepM*	Phosphoenolpyruvate mutase	100	100	0.0004
58227_*Hydroxypyruvate_reductase*	NAD(P)-binding domain-containing protein	75	100	0.0059
58228_*argC_1*	N-acetyl-gamma-glutamyl-phosphate reductase	75	100	0.0059
58229_*lgoD_1*	Alcohol dehydrogenase catalytic domain-containing protein	75	100	0.0059
58230_*Iron_containing_alcohol dehydrogenase*	Iron containing alcohol dehydrogenase	75	100	0.0059
58231_*rhtC_1*	LysE-family-translocator	75	100	0.0059

* Gene annotation was conducted using a blast search against NR database on NCBI.

## Data Availability

Assembly files were submitted to NCBI database under the bio-project PRJNA676043 with accession numbers SAMN16866615 to SAMN16866629.

## Supplementary Materials

The following are available online at https://www.mdpi.com/article/10.3390/microorganisms9081761/s1, Figure S1: Phylogenetic tree based on core-genome alignment of 17 Pantoea stewartii subsp. indologenes strains. Phylogenetic tree was constructed excluding the recombination sites using Parsnp program of Harvest suite. PANS 07-6 was randomly chosen as reference by Parsnp while doing core-genome alignment and identifying SNPs; Figure S2: Sequences per GO terms: Annotations of core, soft-core, shell and cloud genes of Pantoea stewartii subsp. indologenes pan-genome. Panel A-C shows the core genes function to BP, MF and CC; panel D-F shows the function of soft-core genes assigned to BP, MF and CC; panel G-I represent the function of shell genes assigned to BP, MF and CC; panel J-L shows the function of cloud genes assigned to BP, MF and CC; Figure S3: Statistical assessment of annotation differences in Pantoea stewartii subsp. indologenes pan-genome. The panels represent (A) core vs. accessory; (B) soft-core vs. core; (C) shell vs. core; and (D) cloud vs core genes; Table S1: Details of the number of core and accessory genes contributed by each strain; Table S2: List of genes involved in horizontal gene transfer among the twenty-one strains used in this study. Description is the protein coded by these genes and direction shows the gene flow from one strain to another. Depending upon the direction a strain (gene) is defined as a donor or a recipient; Table S3: Mapping and annotation results of donor and recipient genes involved in horizontal gene transfer (HGT); Table S4: Details of GO ids and functional categorization of each protein coding gene sequence involved in HGT. Number of GO ids assigned to each sequence; Table S5: KEGG pathways where the genes featuring in horizontal gene transfer (HGT) are involved; Table S6: List of genes in P. stewartii subs. indologenes that Scoary predicted to be associated with red onion scale clearing phenotype; Table S7: Blast output of Psi genes identified to be associated with red onion scale clearing phenotype showing localisation in PNA-97-1R genome.
