# Supplementary figures and images for: Pan-Genome of Novel Pantoea stewartii subsp. indologenes Reveals Genes Involved in Onion Pathogenicity and Evidence of Lateral Gene Transfer
Source: Microorganisms. 2021 Aug 18;9(8):1761. doi: 10.3390/microorganisms9081761 (PMC8399035; doi:10.3390/microorganisms9081761)

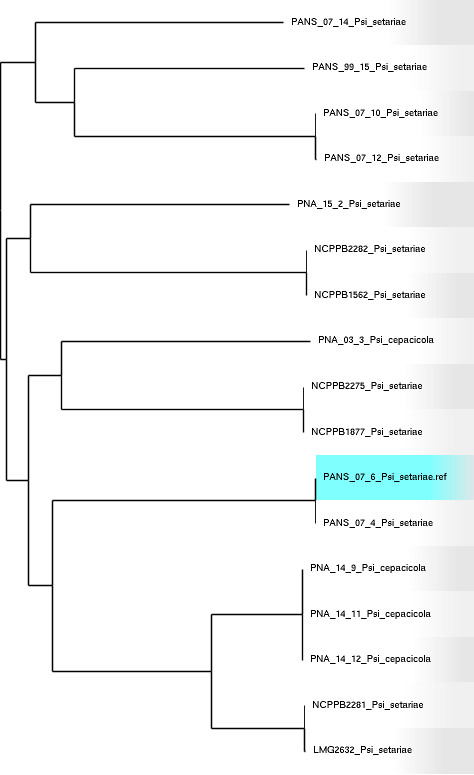

Supplement: Supplementary file 1 [file microorganisms-09-01761-s001.zip › microorganisms-1321841-supplementary/Supplementary/Figure S1.png]

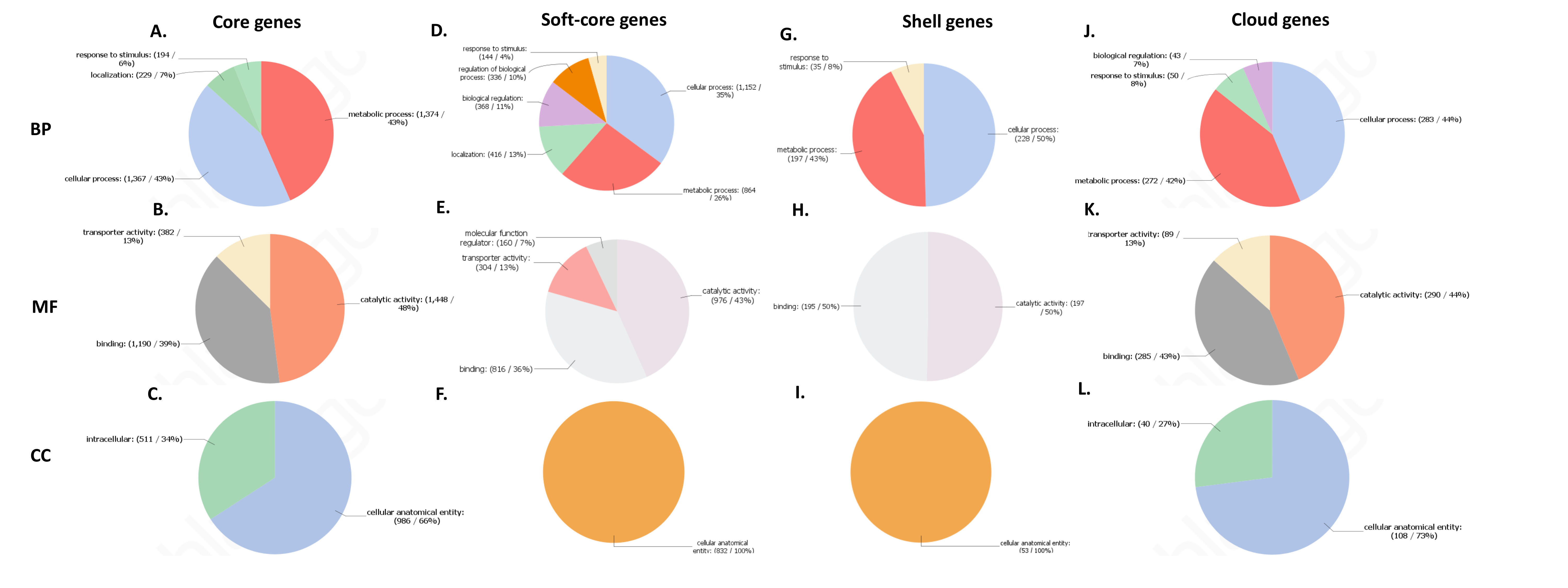

Supplement: Supplementary file 1 [file microorganisms-09-01761-s001.zip › microorganisms-1321841-supplementary/Supplementary/Figure S2.tiff]

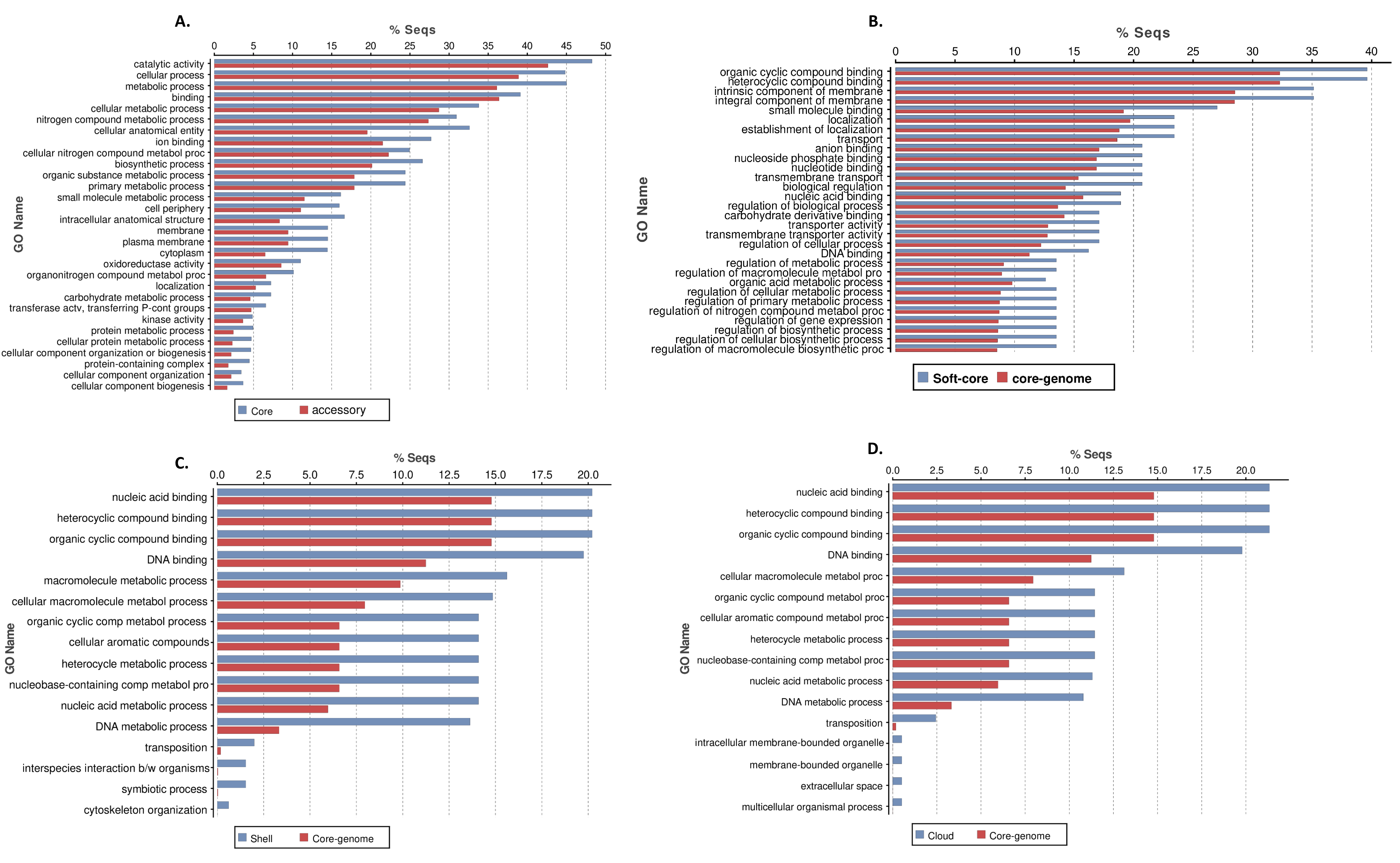

Supplement: Supplementary file 1 [file microorganisms-09-01761-s001.zip › microorganisms-1321841-supplementary/Supplementary/Figure S3.tiff]
